# Open Set Self and Across Domain Adaptation for Tomato Disease Recognition With Deep Learning Techniques

**DOI:** 10.3389/fpls.2021.758027

**Published:** 2021-12-10

**Authors:** Alvaro Fuentes, Sook Yoon, Taehyun Kim, Dong Sun Park

**Affiliations:** ^1^Department of Electronic Engineering, Jeonbuk National University, Jeonju, South Korea; ^2^Core Research Institute of Intelligent Robots, Jeonbuk National University, Jeonju, South Korea; ^3^Department of Computer Engineering, Mokpo National University, Muan, South Korea; ^4^National Institute of Agricultural Sciences, Wanju, South Korea

**Keywords:** open-set recognition, domain adaptation, plant diseases, unknown data, new environments

## Abstract

Recent advances in automatic recognition systems based on deep learning technology have shown the potential to provide environmental-friendly plant disease monitoring. These systems are able to reliably distinguish plant anomalies under varying environmental conditions as the basis for plant intervention using methods such as classification or detection. However, they often show a performance decay when applied under new field conditions and unseen data. Therefore, in this article, we propose an approach based on the concept of open-set domain adaptation to the task of plant disease recognition to allow existing systems to operate in new environments with unseen conditions and farms. Our system specifically copes diagnosis as an open set learning problem, and mainly operates in the target domain by exploiting a precise estimation of unknown data while maintaining the performance of the known classes. The main framework consists of two modules based on deep learning that perform bounding box detection and open set self and across domain adaptation. The detector is built based on our previous filter bank architecture for plant diseases recognition and enforces domain adaptation from the source to the target domain, by constraining data to be classified as one of the target classes or labeled as unknown otherwise. We perform an extensive evaluation on our tomato plant diseases dataset with three different domain farms, which indicates that our approach can efficiently cope with changes of new field environments during field-testing and observe consistent gains from explicit modeling of unseen data.

## Introduction

Plant disease recognition concerns many farmers and researchers in agriculture. Once a plant is affected by diseases, the damage can be easily propagated to the entire crop, causing then several productions and economical losses (Carroll et al., 2017). In conventional farming, crop inspection has been carried out by specialists in the field, which requires a higher level of expertise to understand the complexity of plants and their interactions with factors that cause anomalies (Gelder et al., 2005). This task has been often related to time-consuming, laborious, and subjective. In this regard, over the last few years, several works mainly based on deep learning and computer vision have presented solutions to address this problem using methods such as image classification and object detection (Liu and Wang, 2021). This technology has the potential to reduce the negative impacts of plant diseases by a prompt estimation of the damage using non-intrusive sensors such as RGB cameras (Boulent et al., 2019). Deep learning-based systems have achieved higher recognition and at the same time have contributed with environmental-friendly tools to perform plant state monitoring.

Recent works often rely on classification or detection systems that distinguish between diseases in real-time for various types of crops. Classification methods predict the type of disease using the features of the whole input image, while detection methods estimate both localization and type of disease. These systems have achieved higher performance when the trained models are evaluated in the same or at least similar farm conditions (Ferentinos, 2018). However, a weak performance can be obtained when a model has been trained on a particular dataset (source domain) but evaluated in data from new farms or under different conditions (target domain). This problem has been mainly associated with the capacity of deep neural networks to generalize well in the presence of domain shift, which frequently happens when a system is exposed to limited information provided by datasets that are practically inadequate to cover the large variety of target domains. Domain shift, in this case, is affected by different visual appearances, encouraged by the types of diseases and infection stages, illumination, background, and plant cultivation time.

Existing approaches for plant disease recognition make a closed-set assumption and perform supervised domain adaptation. This assumption is, however, unrealistic as for most real applications, the target domain contains multiple images, but only a small part of it belongs to the classes of interest. The universe of plant diseases is limited to those that have been included in the model during training. Also, this type of domain adaptation requires labels for the new data. In practice, much of this information is novel to the system and is often associated with one of the source categories, which could lead to wrong predictions during inference. This problem is frequently also related to the training data, as it is difficult to obtain and scarce (Barbedo, 2018). Although there are currently annotated datasets available, the collected images generally differ from the type of data that a system should process when it is applied to the real world. Moreover, domain shift can be particularly distinguishable on data collected across farms, where a subset of classes representing the positives for the source domain can be changed, then some existing classes can be disappeared or some new classes could emerge. Therefore, we need to take the “open set” concept for the system to recognize these new classes as an “unknown class” rather than assigning them as one of the existing classes.

**FIGURE 1:**
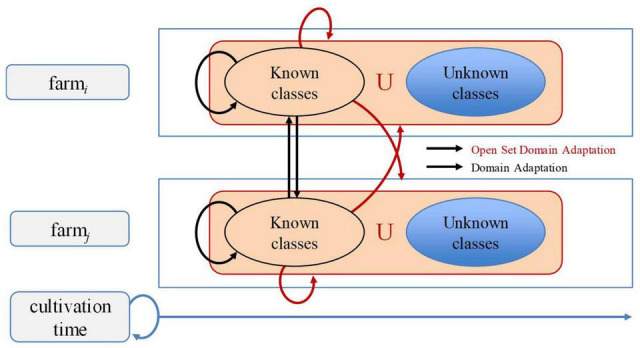
Recognition problem of plant diseases with open set domain adaptation. Our framework addresses open set domain adaptation at farm-level and across different farms. Known classes represent existing classes in the source domain, while unknown classes represent non-existing ones. Since data may vary across farms and throughout the cultivation time, many changes could appear while dealing with novel information.

Early works in deep learning have studied the open-set problem, which is concerned with approaches that are aware of unseen data (Prabhu et al., 2019; Geng et al., 2020). In this article, we aim to cope with the gap between the varying characteristics of data used for training and inference, through domain adaptation by transferring data features of the visual classifier from the source domain to the target domain for plant diseases recognition across different farms. Figure 1 illustrates the recognition problem in the context of our work. Our problem space is extended to both domain-level and class-level. Domain-level can be referred to the conditions of the farm, while class-level represents the types of diseases. Classes include several “known” classes and an “unknown” class. Then, the system operates as follows: Given unknown data, it can tell they are unknown, and given same classes from different farms, it can recognize them as the same.

To summarize, the main contributions of this article are as follows:

- We propose an architecture to address the paradigm of novel data for plant disease recognition. Our proposed method leverages the capabilities of our previous works to address more complex challenges of new greenhouse scenarios.
- Our approach allows performing open set self and across domain adaptation between different greenhouse farms evaluated in our tomato diseases dataset. Particularly, we study domain adaptation using data collected at three different farms. All claims are experimentally evaluated.
- We provide a set of guidelines to analyze the logic behind the recognition of novel data. This approach can allow us to make the system more adaptable to real-world environments.
- We provide theoretical insight and empirical evaluation to demonstrate the capabilities of the proposed system to enhance the performance of plant disease detectors.

The remainder of the article is introduced as follows. Section “Related Works” presents an analysis of related works in open set recognition and plant diseases recognition. Section “Open Set Self and Across Domain Adaptation for Plant Disease Recognition” describes the core of our open-set domain adaptation approach. Section “Experimental Results” shows the experimental settings and results. Finally, Section “Conclusion” concludes the article and presents some guidelines for future works in the field.

## Related Works

In this section, we briefly review recent works related to the proposed approach. According to the constraints of the recent advances in plant disease recognition, these methods fall mainly into closed set domain adaptation. In this direction, open set domain adaptation is referred to as a potential solution to overcome these issues, as a more robust system that can address challenges in real farm conditions.

### Plant Disease Recognition

Plant disease recognition focuses on the estimation of symptoms that occur in the plants due to disease contamination and cause risks to the crops. Recent advances have shown prominent results to address the problems of plant diseases using non-destructive media such as images (Lee et al., 2020). This research field has been considerably developed throughout recent years. Solutions in this area mainly fall into two categories: classification and detection. Classification methods take advantage of the feature distribution of images with a unique label to provide the type of disease in the plant. Early works in the area used Convolutional Neural Networks (CNN) to extract features from images and subsequently classify them into different categories (Mohanty et al., 2016). This idea has been applied to various types of crops such as tomato (Fuentes et al., 2017a), cassava (Ramcharan et al., 2017), grapes (Liu et al., 2020), strawberry (Xiao et al., 2020), among others. However, limitations in this concept rely, for instance, on situations when multiple diseases appear in the same sample or the type of affection has a local or global influence in the plant. In this case, although classification-based methods are simpler to develop and lighter in terms of computational cost, they could fail when applied to real scenarios with varying environmental conditions.

Methods based on detection, on the other hand, synthesize samples more objectively by providing the type of disease and location in the image through the class probability and the bounding box information, respectively. In our previous work (Fuentes et al., 2017b), we presented a baseline framework based on deep learning that can detect 10 types of diseases and pests in tomato plants. In Fuentes et al. (2018), we extended (Fuentes et al., 2017b) and proposed a technique called “refinement filter bank” to cope with the problems of class imbalance and false positives. Recently detection-based recognition has been also applied to other types of crops and diseases. Liu and Wang (2020) proposed a method to detect tomato gray leaf spots using a network based on YOLO-v3. Sharpe et al. (2020) uses YOLOv3 to perform goosegrass detection in strawberry and tomato plants. Kim et al. (2020) proposed a two-stage cascade disease detection model applied to strawberry plants. Afonso et al. (2020) extended the use of deep learning for tomato fruit detection and counting in greenhouses. Fuentes et al. (2019) addressed the problem by combining bounding box information with a text description.

Although both classification and detection strategies have achieved higher performance when trained and evaluated in the same or similar field conditions with defined classes. The situation turns out to be more complex, such as when a system is applied to real-field conditions, an unknown world of objectives could appear in the scene. In this case, a weak performance could be observed, and the problem can be associated with either the capacity of the network to generalize well in the presence of domain shift or the dataset could not cover the large variety of target domains. A system is then severely affected by visual variations and other objects in the scene. Therefore, our proposed research addresses these issues through open set domain adaptation.

### Open Set Domain Adaptation

The interest in studying domain adaptation techniques for computer vision problems has been recently increased as a way to address realistic problems that can be faced in various applications. In real-world recognition tasks, which are limited by various factors, it is usually difficult to collect training samples that cover all types of variations. A more realistic scenario is, therefore, to treat the problem as an open set recognition approach. Scheirer et al. (2013) and Geng et al. (2020) define some basic categories such as “known-known classes (KKCs),” “known unknown classes (KUCs),” “unknown known classes (UKCs),” and “unknown-unknown classes (UUCs).” Depending on the application, these terminologies could be adapted to classification, anomaly detection, one-shot or few-shot learning, zero-shot learning, and open set recognition, where the goal of the last one, is to identify known classes and reject unknown classes.

Differently from closed-set domain adaptation which focuses on mitigating the impact of the domain gap between source and target domains using mainly feature adaptation (Long et al., 2015; Ganin et al., 2017) and generative models (Hoffman et al., 2018; Russo et al., 2018), in open set domain adaptation an incomplete knowledge of the world is presented during training, and unknown data can be submitted during testing, requiring the classifier to effectively not only classify the known categories but also deal with the unseen ones.

Based on the above concept, several strategies have been recently proposed to deal with the issues associated with open set domain adaptation aiming to compensate the domain shift between the source and target datasets (Perera and Patel, 2019; Busto et al., 2020) studied transfer learning for multiple class novelty detection using the knowledge of external datasets to learn negative information of objects that fall outside of the known training data. More relevant to our work, Busto and Gall (2017) explores the field of domain adaptation in open set where only a few categories of interest are shared between the source and target data. In this approach, unknown data is presented at both source and target datasets. Saito et al. (2018), on the other hand, utilized adversarial training to extract features that separate unknown targets from known target samples. Differently from Busto et al. (2020), this method has access to only known source samples and unlabeled target samples for open set domain adaptation. You et al. (2019) introduced “Universal Domain Adaptation (UDA)” which requires no prior knowledge on the label sets. UDA uses a criterion to quantify the transferability of each sample by integrating both the domain similarity and the prediction uncertainty to automatically discover a common label set and also recognizing unknown samples. Also, (Kundu et al., 2020) designed a self-adaptive model that captures the task-specific knowledge from a vendor’s source domain and transfers this knowledge to a client’s target domain, calling this strategy “inheritable models”.

In open set domain adaptation, the level of openness also matters. This is measured by the proportion of unknown classes in the target domain. Scheirer et al. (2013) formalized the “openness” of a problem by considering the number of target classes to be identified, the number of classes used in training, and the number of classes used in testing. Liu et al. (2019) studied the openness of the target domain and presented an approach based on domain adversarial learning called “separate to adapt” using a coarse-to-fine weighting mechanism to separate the samples of unknown and known classes.

In our work, we apply the treats mentioned above and explore methods to learn plant disease diagnosis models with open set domain adaptation. We aim to handle domain-shift among the samples in the dataset to further build a framework capable of dealing with complex cases of real greenhouse scenarios. Also, to the best of our knowledge, there is no available literature on the application of open set domain adaptation in the area of plant disease recognition.

## Open Set Self and Across Domain Adaptation for Plant Disease Recognition

In this section, we describe the assignment of the target samples to categories of the source domain. Also, we introduce the training strategies to address these challenges. Finally, we show how the mapping from the source domain to the target domain is estimated from the previous definitions, and how this influences to label samples that do not match the desired conditions as “unknown.”

### General Settings

**FIGURE 2:**
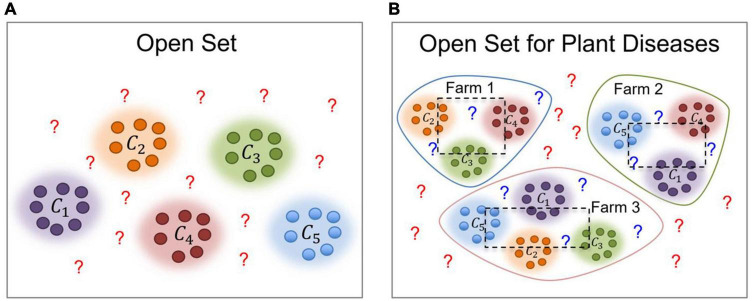
Traditional Open Set vs. Open Set for Plant Diseases. The first one **(A)** contains a universe of data that can be part of the known categories or unknown otherwise. However, in the task of plant diseases **(B)**, we further consider the following conditions: (1) The source where the data has been collected (farm), and (2) the plant states, such as the presence of diseases that can be different at each site. ? represents the unknown data. The dotted boxes represent the portion of data used for training (inside the box) and testing (outside the box) respectively.

Let *y* represent the universe of samples in the dataset that should be diagnosed. Among those samples, data can be either part of specific categories or unknown, as shown in Figure 2. Let *C*_*s*_ = [*c*_1_,*c*_2_,*c*_3_,…,*c*_*n*_] represent a set of *n* classes in the dataset. Open set recognition has been traditionally implemented by dividing the dataset into known and unknown categories. However, in the task of plant diseases, as a real-world application, data is collected not only on a single farm but across multiple farms *L*_*F*_ = [*F*_1_,*F*_2_,*F*_3_,…,*F*_*n*_]. Therefore, although we study the same type of crop, the conditions and plant states can differ from farm to farm. Plants at each farm could be affected by similar or different types of diseases and, also depending on the scenario of application, various conditions such as illumination and background could be observed. Additionally, once deployed, a model may encounter cases that could belong to any of the known categories or declare them as unknown otherwise. In this setting, we want to prevent misdiagnosis of data and instead recommend an additional procedure for those particular samples. Figure 2 illustrates the problem of open set recognition in both, traditional open set and open set for plant disease recognition.

**FIGURE 3:**
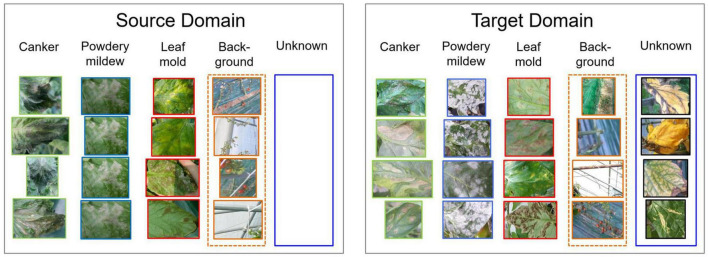
Open set domain adaptation settings. Unknown data is presented only in the target domain. Data from the source and target domains come from different farms, therefore data variation is evident. Additionally, a background class is included to provide contextual information of the greenhouse.

To address open set domain adaptation, we study the actual scenario where unknown data is presented only in the target domain. Figure 3 shows a representation of our open set domain adaptation setting. In plant diseases diagnosis, unknown data correspond to the set of conditions that are rare to find on the farm or novel diseases that are not included in the source dataset utilized for training. In this open set setting, our goal is to cope with diagnosis without using any prior information of unknown data in the source domain. We aim to train a model that is capable of diagnosing data from source classes *C*_*s*_ while avoiding misdiagnosis of data from target classes *C*_*t*_. Additionally, a background class is included in the source domain to add context information of the greenhouse environment.

### Open Set Domain Adaptation

Assuming a dataset with samples belonging to specific classes, in open set domain adaptation (Liu et al., 2019), we have a source domain $D_{s}={\left\{\left(x_{i},y_{i}\right)\right\}}_{i=1}^{n_{s}}$ with *n*_*s*_ labeled samples and a target domain $D_{t}={\left\{\left(x_{j}\right)\right\}}_{j=1}^{n_{t}}$ with *n*_*t*_ unlabeled samples. The source domain consists of a set of classes *C*_*s*_ that are also part of the target domain. However, the target domain is further associated with additional classes that represent unknown data. Then, training domain adaptation can be addressed by assuming a certain level of openness in the dataset. This openness level refers to the relationship of classes used for training, testing, and target. Following (Scheirer et al., 2013), the level of openness in the dataset is introduced as follows:


(1) $$o\,p\,e\,n\,n\,e\,s\,s=1-\sqrt{\frac{2\times \left|\text{training}\,\text{classes}\right|}{\left|\text{testing}\,\text{classes}\right|+\left|\text{target}\,\text{classes}\right|}}$$

This relationship yields the percentage of openness of the problem and fits our application since some classes in the source domain are also part of the target domain. For a fixed number of classes in the source domain, increasing the number of classes in the target domain also increases openness. Different values of openness should be then evaluated to determine the one that perfectly matches the application.

In the context of our application, potential solutions should optimize the recognition of known classes, as well as unknown samples. Different from a closed set setting, in which a new test sample is likely associated as one of the known classes, in open set recognition, a system should be able to label an input as one of the known classes or assign it as unknown otherwise.

### System Overview

Toward open set domain adaptation, first, our system learns to transfer features from the source to the target domain to then addressing the problem associated with domain shift between the datasets used for training and test operations. The main difficulties are, therefore, those associated with the problem of negative transfer and known/unknown separation. Negative transfer is referred to the problem when a system learns to match the whole target domain with the source domain causing also that unknown data also match with the source domain, which consequently leads to negative transfer of information. The other challenge is to separate the known/unknown data in the target domain. A potential solution, in this case, is to adapt the features of the source domain which contains information of the known classes to the target domain.

We follow the facts mentioned above to design our approach for open set domain adaptation toward the recognition of plant diseases. Our proposed architecture consists of two main components: (1) Bounding box detection, (2) Open set domain adaptation.

**FIGURE 4:**
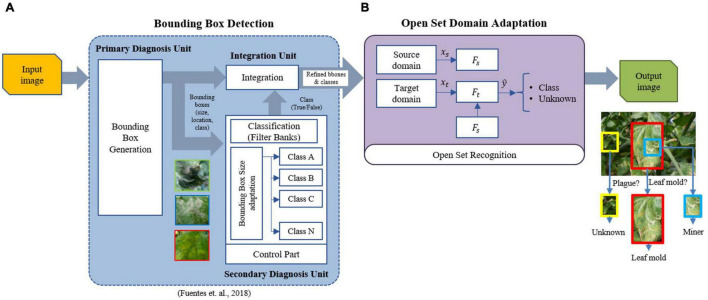
Overall architecture of the proposed work. The system is composed of two blocks: bounding box detector and open set domain adaptation. **(A)** The bounding box detector generates the required bounding boxes using the refinement filter bank method (Fuentes et al., 2018). **(B)** Open set domain adaptation utilizes the set of regions generated by the detector to perform open set recognition by domain adaptation of the source domain (labeled known classes) to the target domain (unlabeled known classes + unknown class). Please see Figure 5 for a detailed description of the components in **(B)**.

Figure 4 presents a general overview of the proposed framework. For each input image, the function of the bounding box detector is to obtain the bounding boxes and corresponding classes of the regions of interest containing plant diseases. Then, the domain adaptation unit learns to assign a target sample to either its respective known class or unknown otherwise. The constraints in this operation include those based on the distance measure to increase the robustness of the system concerning various types of data that can be found in real greenhouse scenarios. Finally, an output image shows the detected regions. We describe in detail each unit of the system below.

#### Bounding Box Detection

The bounding box detector is based on our previous work for plant disease recognition using a refinement filter bank (Fuentes et al., 2018). This architecture utilizes the capabilities of a detector to generate the corresponding Regions of Interest (ROIs) that contain the location and type of diseases. The promising ROIs are then used as input to the filter bank for verification. In this part, misclassified samples are filtered out by training independent CNN classifiers for each class. The main objective of this framework is to determine whether a sample corresponds effectively to the detected category (True) or not (False) otherwise. The output of this block is a set of refined bounding boxes. Therefore, we found that this architecture is suitable to achieve the function of the bounding box detector in the proposed approach. Please refer to Fuentes et al. (2018) for more detail on the characteristics of the detector.

#### Open Set Domain Adaptation

This is the core of the proposed research at which we address the problems associated with domain shift between data used as source and target for the training and test operations (See purple block in Figure 4). Our work is particularly focused on associating a subset of the target samples to the known classes of the source domain or as an unknown class, and computing a representation from the source domain to the target domain by minimizing the distances of the corresponding samples.

**FIGURE 5:**
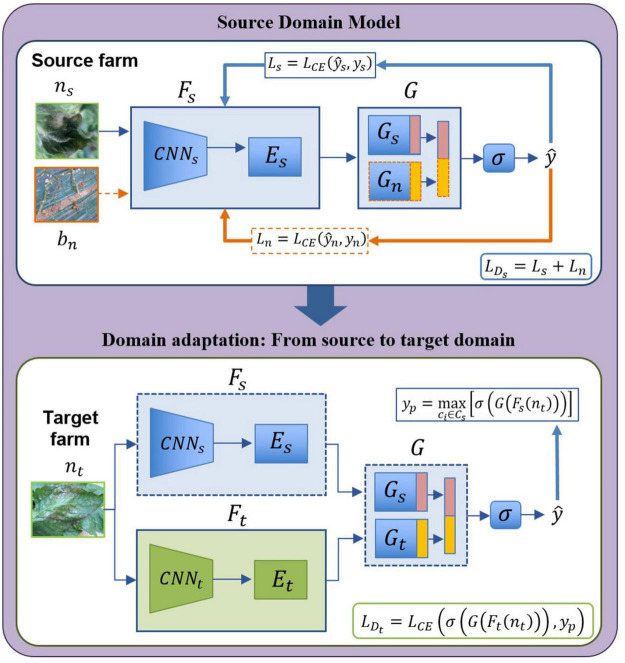
Open set domain adaptation module. “Source farm” represents the data (labeled) of the environments used for training the source domain model. Data (unlabeled) for the target domain comes from a different “Target farm” to test the model on unseen samples which belong to either one of the known classes or unknown class.

The architecture for open set domain adaptation is summarized in Figure 5. The proposed design is carried out based on the following two steps:

- *Source domain model:* This model captures the features of known classes distributed in the source domain. Given a set of classes *C*_*s*_ in the source domain $D_{s}={\left\{\left(x_{i},y_{i}\right)\right\}}_{i=1}^{n_{s}}$ with *n*_*s*_ labeled samples, the feature extractor *F*_*s*_ includes a backbone *CNN*_*s*_ and fully connected layers *E*_*s*_. The classifier *G* contains two parts: a source classifier *G*_*s*_ for the known classes, and a control classifier *G*_*n*_ to add contextual information *b*_*n*_. The control samples represent regions that do not correspond to any type of disease, but parts of the farm background to add contextual information to the system (See the background class in Figure 3). The output ${\hat{y}}_{s}$ is obtained by concatenating the features of *G*_*s*_ and *G*_*n*_ followed by a softmax function σ that categorizes samples of the source domain while including also negative information for class separability. The response of *G*_*s*_ (source data) is maximized, by also maximizing the response of *G*_*n*_ with respect to the background samples. Training the source domain model involves using a cross-entropy loss function in two steps: First, $L_{s}=L_{C\,E}\,\left({\hat{y}}_{s},y_{s}\right)$ is used to train the source data *D*_*s*_, where ${\hat{y}}_{s}=\sigma \,\left(G_{s}\,\left(F_{s}\,\left(n_{s}\right)\right)\right)$, then by freezing the features of *CNN*_*s*_, negative instances *D*_*n*_ are generated from *b*_*n*_ and training *E*_*s*_,*G*_*s*_,*G*_*n*_ using $L_{n}=L_{C\,E}\,\left({\hat{y}}_{n},y_{n}\right)$, where ${\hat{y}}_{n}=\sigma \,\left(G_{n}\,\left(E_{s}\,\left(b_{n}\right)\right)\right)$. The total loss in the source domain is obtained as follows,
(2) $$L_{D_{s}}=L_{s}+L_{n}$$
  where *L*_*s*_ and *L*_*n*_ represent the source and negative loss, respectively. Once the source domain is trained, the system owns features from both source data and negative data.
- *Domain adaptation*: By domain adaptation, we aim to cover the gap of the domain-shift between the source and target data. Like the source model, domain adaptation starts with a given set of classes $D_{t}={\left\{\left(x_{j}\right)\right\}}_{j=1}^{n_{t}}$with *n*_*t*_ unlabeled samples of the target farm. In this case, features from the source domain model (source farm) are utilized as an initial point to transfer information from known labeled classes and background class to the target domain (target farm), then *F*_*s*_ and *G* are frozen. An input data *n*_*t*_ goes in two directions: first, it is passed through *F*_*s*_ and *G* using finetuning. Then, features are obtained by *F*_*t*_ from *CNN*_*t*_ and *E*_*t*_ to further, by classification in *G*_*t*_ generating the features of the target domain. The output ${\hat{y}}_{p}$ is then obtained by concatenating the responses of *G*_*s*_ and *G*_*t*_ followed by the softmax function σ. At this point, the task is to maximize the probability in case the sample belongs to one of the known classes by $y_{p}=\max_{c_{i}\in C_{s}}\left[\sigma \,\left(G\,\left(F_{s}\,\left(n_{t}\right)\right)\right)\right]$. Training the domain adaptation model aims to minimize the following function:
(3) $$L_{D_{t}}=L_{C\,E}\,\left({\hat{y}}_{p},y_{p}\right)$$

where, ${\hat{y}}_{p}=\sigma \,\left(G\,\left(F_{t}\,\left(n_{t}\right)\right)\right)$. Minimizing this function allows us to efficiently determine if a sample belongs to either one of the known classes or unknown class in the target domain.

Finally, the output of the system is an image with the detected plant diseases if they coincide with any of the corresponding known classes of the source domain. Otherwise, these regions are labeled as unknown.

### Evaluation Metrics

#### Bounding Box Detector

In line with (Fuentes et al., 2018), we evaluate the performance of the bounding box detector using the following metrics:

- *Intersection-over-Union metric (IoU):* We utilized a threshold of 0.5 to capture true positive detections generated by the model, as:
(4) $$I\,o\,U=\left|\frac{A\cap B}{A\cup B}\right|$$
  where A and B represent the ground-truth and predicted box, respectively.

- *Mean Average Precision score (mAP):* mAP is the area under the precision-recall curve calculated for all classes.
(5) $$A\,P=\frac{1}{11}\,\sum_{r\in \left[0,0.1,\ldots ,1\right]}P_{i\,n\,t\,e\,r\,p}\,\left(r\right)$$
(6) $$P_{i\,n\,t\,e\,r\,p}\,\left(r\right)=\max_{\tilde{r}:\tilde{r}\geq r}p\,\left(\tilde{r}\right)$$
  where, $P_{i\,n\,t\,e\,r\,p}\,\left(\tilde{r}\right)$ is the maximum precision for any recall values greater than *r*, and $p\,\left(\tilde{r}\right)$ is the measured precision at recall $\tilde{r}$.

#### Domain Adaptation

Following (Saito et al., 2018), the usual metrics adopted to assess the performance of the open set domain adaptation framework are the normalized accuracy over the known classes OS*, and the accuracy of the unknown class UNK. These two metrics are usually combined in OS as a measure of the overall performance. Additionally, following (Bucci et al., 2020), we further evaluate the performance using the harmonic mean of OS* and UNK (HOS). HOS provides a high score only if the algorithm performs well both on known and on unknown samples, independently of the number of classes K. Therefore, using HOS instead of a simple average penalizes large gaps between OS* and UNK. The evaluation metrics are presented below:

- Normalized accuracy over the known classes (OS*):

(7) $$O\,S^{*}=\frac{1}{K}\,\sum_{k=1}^{K}A\,c\,c_{k}$$

- *Normalized accuracy over all classes including the unknown (OS):*

(8) $$O\,S=\frac{1}{K+1}\,\sum_{k=1}^{K+1}A\,c\,c_{k}$$

where *k* represents the number of known classes.

- Harmonic mean of OS* (HOS):

(9) $$H\,O\,S=2\,\frac{O\,S^{*}\times U\,N\,K}{O\,S^{*}+U\,N\,K}$$

## Experimental Results

In this section, we evaluate the performance of the proposed open set domain adaptation approach for plant disease recognition. We design the experiments to support our claims, then: (1) we provide a framework for plant diseases recognition that can operate in real greenhouse scenarios, (2) we address the problem of domain shift between the datasets used for training and testing, respectively, (3) our model can efficiently transfer the features of the source domain to the target domain and perform recognition in unseen data of different farm environments.

### Dataset Settings

We carried out experiments and validate the performance of our approach on our tomato plant diseases dataset (Fuentes et al., 2017b,^2018^). This dataset has been collected and updated over the last 5 years at different locations and farms in South Korea using various types of camera devices. It includes several variations associated with real scenarios of greenhouses such as illumination, scales, sizes, and plant states with various infection stages. To validate the performance of the proposed approach, we selected three specific farms with different conditions and data variations.

The dataset consists of 12 types of tomato plant diseases, pests, and physiological disorders, plus two additional classes that contain healthy leaves and regions of the background, respectively. Details on the dataset are presented in Table 1. The number of samples represents the annotated bounding boxes before and after data augmentation. We used extensive data augmentation such as geometric and intensity transformations to increase the size of the dataset as in Fuentes et al. (2017b).

**TABLE 1:** Tomato plant diseases dataset and data availability by farm for open set domain adaptation.

No.	Class	Farm data availability			Number of annotated bounding boxes	Number of bounding boxes after data augmentation
		Farm 1	Farm 2	Farm 3		
1	Leaf mold	×	×		7,178	35,890
2	Gray mold		×	×	523	2,615
3	Canker	×	×		618	3,090
4	Powdery mildew	×		×	6,277	31,385
5	Tomato yellow leaf curl virus (TYLCV)			×	12,918	64,590
6	Healthy	×	×	×	12,252	61,260
7	Background	×	×	×	2,469	12,345
8	Tomato chlorosis virus (ToCV)	×		×	4,190	20,950
9	Plague		×		598	2,990
10	Miner	×		×	2,328	11,640
11	Whitefly	×	×		1,701	8,505
12	Whitefly egg	×	×		6,314	31,570
13	Magnesium deficiency			×	584	2,920
14	Physical damage	×	×	×	1,767	5,835
	TOTAL				59,717	295,585

#### Farm Data Distribution

**FIGURE 6:**
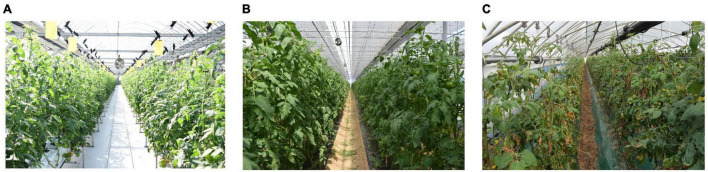
Representation of the greenhouses used to collect images of tomato plant diseases and pests. Each place has different conditions and visual variations. **(A)** Farm 1, **(B)** Farm 2, and **(C)** Farm 3.

To make our dataset able to be used for domain adaptation such as the representation shown in Figure 2B, we considered the case of data collected at three different locations and farms described as *F*_*1*_, *F*_*2*_, *F*_*3*_. This is a particular case of domain adaptation of various scenarios toward building a more robust system by taking the visual characteristics from one place to another. Here, we assume that the conditions of each greenhouse farm are different, for instance, in terms of illumination, materials of the structure, and surrounding objects. Also, because some diseases or pests can appear commonly throughout the farms, while others appear only in some of them. A representation of the farms used for data collection is presented in Figure 6.

During our study, we used data collected on both modern and traditional greenhouse structures. Modern greenhouses appear to have clear backgrounds, hydroponic crop management, and automatic control of the cultivation processes, while more traditional greenhouses tend to have darker backgrounds due to the structure materials, and also plants are cultivated in the soil. This, consequently, can be reflected in the types of diseases and pests that can be found on each farm.

#### Types of Diseases and Pests

For our study, we selected the most common tomato plant diseases and physiological disorders that occur in Korean farms such as leaf mold, gray mold, canker, powdery mildew, tomato yellow leaf curl virus (TYLCV), tomato chlorosis virus (ToCV), plague, magnesium deficiency, and physical damage. Also, some pests like miner, whitefly, whitefly egg. Additionally, we included another class of healthy leaves and a background class that is particularly used for adding contextual information about the visual characteristics of the greenhouse and surrounding areas of the plant. The availability of data by farm and class is presented in Table 1.

#### Source and Target Datasets

Considering the data availability for each class, to build up the source and target datasets for domain adaptation, our experiments are focused on the following combinations across three different farms: F1-to-F2, F1-to-F3, F2-to-F1, F2-to-F3, F3-to-F1, F3-to-F2. Correspondingly, we train the source domain using data from one farm and transfer its features to the other domain.

#### Known and Unknown Classes

To assign, the number of classes that are used for training and testing the source and target domains, respectively, we used the formula described previously in Eq. 1 with different levels of openness. In this way, we evaluate the response of the system to the percentage of known and unknown classes. We used different values of openness such as 0, 33, 62, and 73%, respectively. 0% represents the case of closed-set recognition, which is the case when we apply the same number of classes for training and testing. Table 2 shows the calculated openness level based on the number of training, testing, and target classes.

**TABLE 2:** Calculation of the openness level for domain adaptation based on Eq. 1.

Number of classes			Openness (%)
Training	Testing	Target	
14	14	14	0
4	9	9	0.33
2	14	14	0.62
1	14	14	0.73

### Implementation

We applied the settings mentioned above for training and testing the model across three different domain farms. The complete model is implemented in the following two stages:

#### Bounding Box Detector

We used the architecture of Fuentes et al. (2018) as the bounding box detector for ROI recognition introduced in the section “System Overview.” As the core implementation, the model consists of a primary diagnosis function implemented based on Faster R-CNN with VGG-16 network as the feature extractor. However, differently from the original version, here, we utilized a ResNet-50 with Feature Pyramid Network (FPN) as the feature extractor, because of its potential for detecting objects at various scales. We named this version “Filter Banks v2” in Table 3. The secondary diagnosis unit and integration units are implemented using the same configuration as the original version. Table 3 presents a performance comparison of existing methods for bounding box detection.

**TABLE 3:** Performance of the bounding box detector (mAP).

Class	FRCNN-VGG-16 (Fuentes et al., 2017b)	SSD-ResNet-50 (Fuentes et al., 2017b)	RFCN-ResNet-50 (Fuentes et al., 2017b)	Filter Bank-ResNet-50 (Fuentes et al., 2018)	Filter Bank v2 – ResNet-50 FPN (Proposed)
Leaf mold	0.8910	0.8421	0.8591	0.9312	**0.9637**
Gray mold	0.7935	0.7745	0.7810	0.8823	**0.9004**
Canker	0.8400	0.8300	0.8562	0.9451	**0.9524**
Powdery mildew	0.6321	0.8145	0.7748	**0.9745**	0.9619
Tomato yellow leaf curl virus (TYLCV)	0.8500	0.7680	0.8610	0.9498	0.9641
Healthy	0.8913	0.8540	0.8875	**0.9614**	0.9600
Background	0.9005	0.8841	0.8921	**0.9450**	0.9296
Tomato chlorosis virus (ToCV)	0.9111	0.8600	0.9098	0.8745	**0.9433**
Plague	0.8510	0.8409	0.8641	0.9710	**0.9745**
Miner	0.7856	0.7963	0.8447	0.8143	**0.9310**
Whitefly	0.8301	0.8298	0.8492	0.9580	**0.9604**
Whitefly egg	0.7800	0.7511	0.7720	0.9213	**0.9314**
Magnesium deficiency	0.7824	0.7892	0.7545	**0.9821**	0.9796
Physical damage	0.7548	0.6301	0.6847	0.8946	**0.9145**
mAP	0.82095	0.8046	0.8279	0.9289	**0.9476**

*Values in bold represent the best performance obtained for each class with respect to the applied models.*

Our Filter Bank v2 (proposed) detector provides an accurate yet efficient alternative to obtain refined regions while avoiding the false positives in the final prediction. We trained and validated the detector on the entire tomato plant diseases dataset to obtain the set of bounding boxes and their corresponding classes. We used an Intersection over Union (IoU) threshold of 0.5. A representation of the bounding boxes generated by the detector can be seen in Figure 3.

#### Open Set Domain Adaptation

Once the regions of interest corresponding plant diseases have been obtained from the output of the detector, we defined the known and unknown categories to be used as part of the source and target domains, also taking into account the information of the farm. We used an ImageNet pre-trained ResNet-50 model as the base CNN feature extractor (Figure 5), where the last fully connected layer was replaced with the task-specific FC layers to parameterize the classifier. We finetuned the pre-trained layers and trained the newly added layers where the learning rate is adjusted along the training process. Domain adaptation has been evaluated across multiple combinations of the three farms utilized in this study. Evaluation results are presented in terms of OS*, OS, and HS introduced earlier in Eqs. 7, 8, and 9, respectively. Details on training and testing the source and target domains are presented below:

- **Source domain**: We trained the model on the source dataset with known classes. We used a batch size of 64 for 30,000 iterations. Validation is performed every 30 iterations and the learning rate has been adjusted to 0.001.
- **Target domain**: Like training the source domain, we used a batch size of 64 to train the target domain with the known and unknown classes for 30,000 iterations. However, validation is performed every 500 iterations and the learning rate has been adjusted to 0.0001.

### Quantitative Results

We evaluated the performance of the bounding box detector and the open set domain adaptation module across the combinations of three farms, with the openness levels presented in Table 2. The results of the experiments are presented as follows.

#### Performance of the Bounding Box Detector

In this experiment, we evaluate the performance of the bounding box detector compared to other existing methods, and report the mAP calculated with Eq. 5. As mentioned earlier, the core of the bounding box detector is based on our previous work using filter banks for plant disease recognition (Fuentes et al., 2018). The only difference with the current detector is the application of the feature extractor ResNet-50 with a feature pyramid architecture (FPN) to make the system able to distinguish object at various scales, especially the small-scale objects such as miner, whitefly, and whitefly eggs. We trained the detector to recognize bounding boxes and types of diseases and obtained an outstanding performance of mAP 94.76% compared to other existing methods used for the similar task, which represents an improvement of 1.8% compared to Fuentes et al. (2018) using the current dataset. Table 3 presents the results of this experiment.

#### Accuracy in the Target Domain

##### Accuracy Over the Known Classes (OS*)

In this experiment, we evaluate the performance of domain adaptation from the source to the target dataset over the known classes. To calculate this value, we used the metric introduced in Eq. 7. Table 4 shows the accuracy across the combination of farms using different levels of openness such as 0% which is the case of closed-set, 33, 62, and 73%, respectively. We present the OS* accuracy averaged over three runs on the regions of interest (ROIs) containing plant diseases obtained from the detector.

**TABLE 4:** OS* accuracy (%) of the known classes across the combination of farms, averaged over three runs on the tomato diseases dataset.

Openness (%)^1^	Farms					
	F1-to-F2	F1-to-F3	F2-to-F1	F2-to-F3	F3-to-F1	F3-to-F2
0	94.30	89.45	91.02	92.78	90.01	93.58
33	92.14	84.14	86.98	88.50	83.57	89.80
62	85.85	80.02	83.12	86.01	79.18	80.45
73	73.05	69.48	72.04	78.36	68.44	71.59

*^1^The % of openness is calculated using Eq. 1 as shown in Table 2.*

**FIGURE 7:**
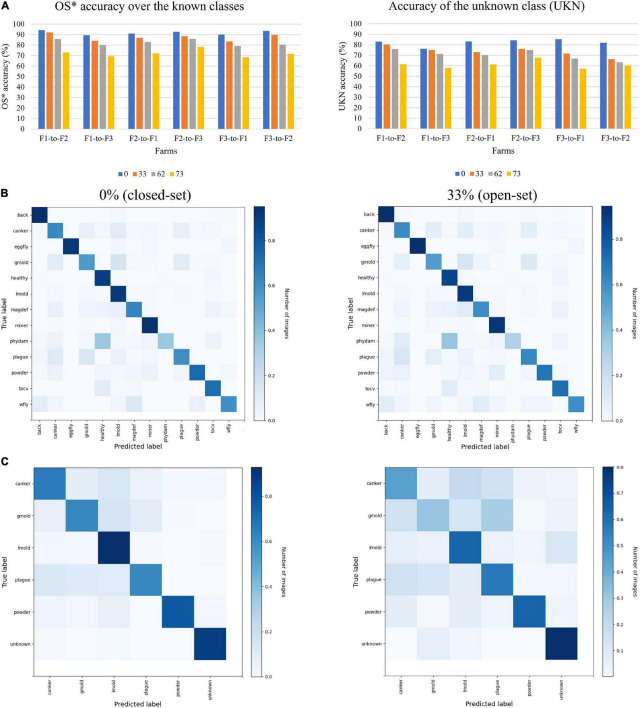
Recognition of known classes vs. unknown class on the target dataset. **(A)** OS* accuracy over the known classes. **(B)** Accuracy of the unknown (UKN) class. Bars represent the openness levels from 0% (closed-set) to 33%, 62%, and 73%. **(C)** Confusion matrix of domain adaptation from Farm 1 to Farm 2 (F1-to-F2) with openness of 0% (closed-set) and 33% for the known classes. **(D)** Confusion matrices for some known classes and unknown class. The label abbreviations represent the following classes: back, background; gmold, gray mold; lmold, leaf mold; powder, powdery mildew; tocv, tomato chlorosis virus; wfly, whitefly; eggfly, whitefly egg; magdef, magnesium deficiency; phydam, physical damage.

The system efficiently recognized samples from the known categories. However, as the level of openness increases, the performance showed some decay in all cases. Among the combinations of farms, we found that F1-to-F3, and F3-to-F1, were the most challenging cases. On one side, Farm 1 (F1), as shown earlier in Figure 6A, represents the case of a modern type of greenhouse with clear background and appropriate control system, while Farm 3 (Figure 6C) shows the case of a more traditional type of greenhouse which is more common to find. Transferring features from one domain to the other domain represents, therefore, an issue as the visual characteristics of both farms are different since we get access to more data variations. Differently, transferring features from F1-to-F2 and F2-to-F3 showed the best results, respectively. Compared to closed-set recognition (0%), where we obtained an accuracy of 94.30% for the best case on F1-to-F2, and similarly, for the other cases, open-set still shows weakness. Conceptually at closed-set recognition, we used the same number of classes for training and testing, that is 0% openness. Figure 7A shows a visualization of the accuracy of known classes. There, we can evidence the performance difference of closed-set recognition against open-set recognition with different openness values.

##### Accuracy Over All Classes Including Unknown (OS)

In this experiment, we evaluate the capability of the model to address the recognition of data from both known classes and unknown in the target dataset. To calculate this value, we used the metric presented in Eq. 8. Table 5 shows the OS accuracy averaged over three runs on the ROIs (target dataset) obtained from the detector. For evaluation, given unknown data, the system should tell they are unknown. Similarly, given know data they should be recognized as the same. This is a representation of the particular case where the system has to deal with new types of diseases or disorders once applied to a new greenhouse environment different from the ones used for training.

**TABLE 5:** OS accuracy (%) for all classes including unknown across the combination of farms, averaged over three runs on the tomato diseases dataset.

Openness (%)^1^	Farms					
	F1-to-F2	F1-to-F3	F2-to-F1	F2-to-F3	F3-to-F1	F3-to-F2
0	93.07	88.75	89.00	90.56	89.01	92.60
33	86.48	82.48	84.47	83.04	79.63	87.98
62	79.40	76.01	77.45	79.70	74.08	75.78
73	62.31	60.01	63.04	65.48	61.13	59.13

*^1^The % of openness is calculated using Eq. 1 as shown in Table 2.*

In this case, recognizing unknown data over the wide spectrum of variations presented in the target dataset is more challenging. Unknown data could include samples that do not match any of the known classes and are presented only in the target domain. The results demonstrate a further performance decay at a larger level of openness, but the system is still able to recognize unknown data.

As in the previous experiment, a closed set configuration generates better performance than the open set. However, what we intend to demonstrate exactly with our work is a more realistic scenario to improve the ability of a model to be able to recognize new unknown classes. This could be the case when the system finds unknown or new diseases on new farms. Therefore, the level of openness gives the idea of this scenario.

##### Harmonic Mean of OS*

As presented earlier in the section “Quantitative Results” and Eq. 9, HOS represents a more realistic estimation of the recognition problem, as it provides a high score only if the algorithm performs well both on known and on unknown samples, independently of the number of classes. Therefore, using HOS penalizes large gaps between OS* and UNK. Following (Bucci et al., 2020), we compare the performance of the model using three metrics.

Table 6 presents the comparison of the calculated accuracies. By analyzing these three metrics, we can notice the performances difference between the recognition of known classes against the unknown class in all cases. Recognizing the unknown class is more challenging, and therefore, in this type of open-set domain adaptation systems, it is important to independently measure the recognition of the unknown class to understand the capabilities of the model in this recognition task. Additionally, HOS reflects better the open set scenario for both known classes and unknown class.

**TABLE 6:** OS, UNK, and HOS accuracies (%) averaged over three runs on the tomato diseases dataset.

Open (%)^1^	Farms																	
	F1-to-F2			F1-to-F3			F2-to-F1			F2-to-F3			F3-to-F1			F3-to-F2		
	OS^*^	UNK	HOS	OS^*^	UNK	HOS	OS^*^	UNK	HOS	OS^*^	UNK	HOS	OS^*^	UNK	HOS	OS^*^	UNK	HOS
0	94.30	83.05	88.32	89.45	76.15	82.27	91.02	83.25	86.96	92.78	84.32	88.35	90.01	85.38	87.63	93.58	82.03	87.43
33	92.14	80.42	85.88	84.14	75.20	79.42	86.98	73.10	79.44	88.50	76.04	81.80	83.57	71.65	77.15	89.80	66.25	76.25
62	85.85	76.01	80.63	80.02	71.43	75.48	83.12	70.26	76.15	86.01	75.15	80.21	79.18	67.05	72.61	80.45	63.46	70.95
73	73.05	61.48	66.77	69.48	58.09	63.28	72.04	61.43	66.31	78.36	67.72	72.65	68.44	57.32	62.39	71.59	60.18	65.39

*^1^The % of openness is calculated using the Eq. 1 as shown in Table 2.*

This experiment also validates the domain adaptation across the three farms. We evidence that transferring features from F1-to-F2 and F2-to-F3 farms, results in higher accuracy than the other cases. However, the performance decays as the openness value is increased. Figure 7 shows a representation of the accuracy differences between OS* accuracy for the known classes (Figure 7A) against the accuracy of the unknown class (Figure 7B) at different openness values. Similarly, we can observe the performance of closed-set (0% openness) against open-set recognition (33, 62, and 73% openness). The performance of known classes is higher than the unknown. Also, in both cases, the performance decays as the level of openness is increased. This result further demonstrates the complexity of transferring features across domains with different conditions and data.

To further validate the performance of the proposed approach, we present the confusion matrices of domain adaptation from one farm to another. We selected Farm 1 to Farm 2 (F1-to-F2) to visualize the changes for two values of openness from 0% representing the closed-set case, and 33% representing the open-set case, respectively. First, Figure 7C presents the performance in the known classes. Here, we can observe the types of diseases and their influence in the final prediction as the level of openness increases. Generally, healthy and leaf mold classes obtained higher recognition priority as compared to the rest of the classes. Less influential, but still recognized, are the rest of the classes. Additionally, we can visualize that as the level of openness increases, some of the classes show slight levels of confusion with the other classes.

Figure 7D presents the confusion matrices for the cases of the unknown class along with the known classes. Here, we can observe the tendency of classes that tend to contribute to the recognition of the unknown class. Similarly, as the level of openness increases, samples that do not match with the features of any of the known classes are recognized as unknown. Therefore, we can observe a higher tendency of data to be recognized as unknown.

#### Training and Loss Curves Across Domains and Farms

**FIGURE 8:**
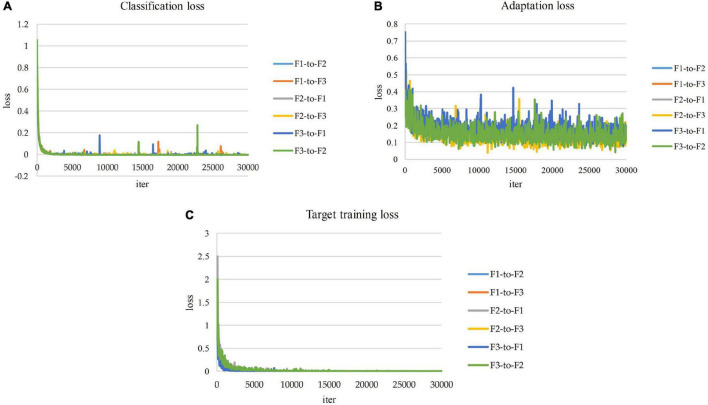
Training losses. **(A)** Source training loss. **(B)** Adaptation loss. **(C)** Target training loss.

Figure 8 presents the resulting training losses and accuracy curves for the implementation of domain adaptation across the three domain farms utilized for our study. The system learned to adapt appropriately from one domain to the other, and data from known classes as well as novel information added in the target domain is effectively recognized.

### Qualitative Results

**FIGURE 9:**
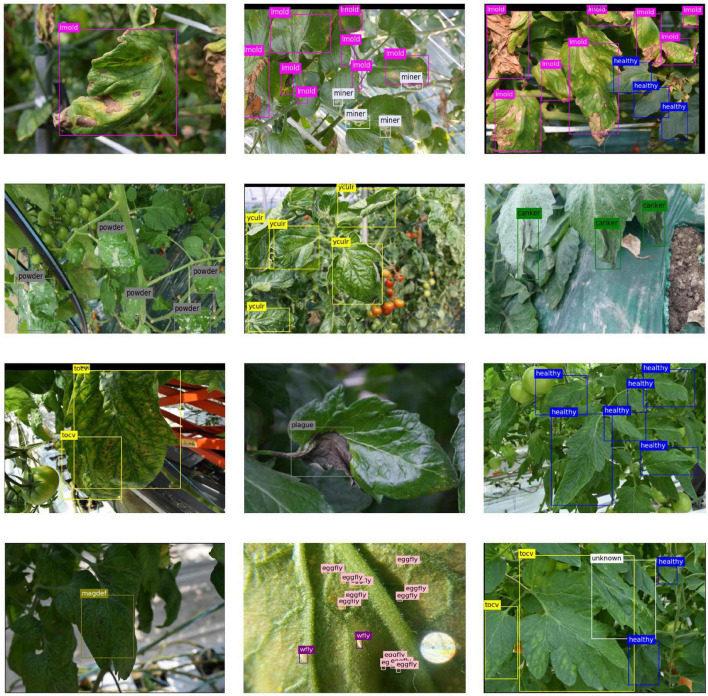
Examples results of known and unknown classes on the tomato plant diseases and pests dataset. To obtain these results, we used an openness level of 33%. The label abbreviations represent the following classes: lmold, leaf mold; powder, powdery mildew; yculr, tomato yellow leaf curl virus (TYLCV); tocv, tomato chlorosis virus; wfly, whitefly; eggfly, whitefly eggs; magdef, magnesium deficiency.

Figure 9 shows some example qualitative results generated by the system on the testing dataset. For this experiment, we used an openness level of 33%. The system satisfactorily detected various types of diseases and physiological disorders that affect tomato plants. Also, in case a region does not match the features of the known classes, it is labeled as unknown. Despite the complexity of the greenhouse environments used for domain adaptation, our approach showed further robustness to address the detection of objects with various scales and damage stages. We believe part of the reason lies in the feature transformation procedure from various domains to feed more features and context information to the system. Specifically, when transferring features from the source domain to the target domain, our feature adaptation implicitly aggregates information from other greenhouse environments, and thus produces stable detection results.

## Conclusion

In this article, we presented an approach for open-set domain adaptation for plant disease recognition to allow existing systems to operate in new environments with unseen conditions and farms. Our system specifically addressed diagnosis as an open set problem by mapping the features of the source and target domains to potentially improve the performance of the known classes while treating changes that happen in real farm conditions as unknown information. The main framework consists of two modules that perform bounding box detection and open set domain adaptation. We performed an extensive evaluation on our tomato plant diseases dataset across three different domain farms. An interesting future direction is, to explore the adaptation of the model to more farms with the possibility of extending the study to more diseases and variations, as well as to other types of crops. Also, improve the potential of the model to compensate for the deterioration in performance due to the level of openness.

## Data Availability Statement

The original contributions presented in the study are included in the article/supplementary material, further inquiries can be directed to the corresponding authors.

## Author Contributions

AF designed the system, performed the experiments, and wrote the manuscript. DP and SY advised in the design of the system and analyzed the strategies to find the best method for efficient plant diseases recognition. TK provided support in the data collection. All authors contributed to the article and approved the submitted version.

## Conflict of Interest

The authors declare that the research was conducted in the absence of any commercial or financial relationships that could be construed as a potential conflict of interest.

## Publisher’s Note

All claims expressed in this article are solely those of the authors and do not necessarily represent those of their affiliated organizations, or those of the publisher, the editors and the reviewers. Any product that may be evaluated in this article, or claim that may be made by its manufacturer, is not guaranteed or endorsed by the publisher.
